# Complexation of Nickel Ions by Boric Acid or (Poly)borates

**DOI:** 10.1007/s10953-016-0555-x

**Published:** 2016-12-17

**Authors:** Anais Graff, Etienne Barrez, Philippe Baranek, Martin Bachet, Pascale Bénézeth

**Affiliations:** 1 EDF R&D – Department of Material and Mechanic of Component, EDF Lab Les Renardières, Avenue des Renardières – Ecuelles, 77818 Moret-Sur-Loing Cedex, France; 2 PPSM, ENS Cachan, CNRS, Université Paris – Saclay, 94235 Cachan, France; 3 EDF R&D, Department Economic and Technical Analysis of Energy Systems (EFESE) – Institut of Research and Development on Photovoltaic Energy (IRDEP), EDF Lab – Chatou, 6 quai Wattier, 78400 Chatou Cedex, France; 4 Géosciences Environnement Toulouse, CNRS, Observatoire Midi-Pyrénées, Université de Toulouse, 14 avenue Edouard Belin, 31400 Toulouse, France

**Keywords:** Aqueous, Complexation, Boric acid, Nickel, Equilibrium constant, First principles, DFT

## Abstract

An experiment based on electrochemical reactions and pH monitoring was performed in which nickel ions were gradually formed by oxidation of a nickel metal electrode in a solution of boric acid. Based on the experimental results and aqueous speciation modeling, the evolution of pH showed the existence of significant nickel–boron complexation. A triborate nickel complex was postulated at high boric acid concentrations when polyborates are present, and the equilibrium constants were determined at 25, 50 and 70 °C. The calculated enthalpy and entropy at 25 °C for the formation of the complex from boric acid and Ni^2+^ ions are respectively equal to (65.6 ± 3.1) kJ·mol^−1^ and (0.5 ± 11.1) J·K^−1^·mol^−1^. The results of this study suggest that complexation of nickel ions by borates can significantly enhance the solubility of nickel metal and nickel oxide depending on the concentration of boric acid and pH. First principles calculations were investigated and tend to show that the complex is thermodynamically stable and the nickel cation in solution should interact more strongly with the ${\text{B}}_{3} {\text{O}}_{3} \left( {\text{OH}} \right)_{4}^{ - }$ than with boric acid.

## Introduction

The primary circuit of a pressurized water reactor (PWR) is chemically conditioned with boric acid as neutron adsorber [1]. Its hydrolysis giving borate ions has a major effect on the pH of the routine power plant cycles. Nevertheless, in certain conditions of temperature and concentration, other species, such as polyborates, can also be formed and need to be taken into account [2, 3]. In addition, the PWR primary circuit is subject to corrosion issues linked to the formation of different oxides, called corrosion products. Some particles of these oxides are released in the primary fluid and may be activated when they pass through the core of the reactor. Even if the quantity of these particles is very low, the radiation dose rates can be strongly influenced by their deposition on the whole primary circuit. Nickel comes from steam generator tubing alloys and is a corrosion product of major importance because of its activation into ^58^Co and its impact on the radioactive contamination of the circuit. Precise knowledge of the solubility of corrosion products containing nickel and how it changes with temperature and chemistry would be valuable to understand the transport of nickel from steam generator tubes to the core. Previous experimental studies have focused on the solubility of nickel oxide and nickel metal at high temperatures and pressures in different media including PWR operating conditions but the results are subject to discrepancies [4–8]. One explanation could be the complexation of nickel ions by (poly)borates, which can increase the solubility of the solid phases.

Two principal studies have focused on the nickel–boron complex formation. Palmer et al. have studied the solubility of nickel hydroxide in different media, including low concentrations of boric acid [6]. The nickel concentrations were found to be higher in boric acid medium than all the remaining data collected in the study. However, no hypothesis was offered to explain that solubility enhancement, especially because results were in the reverse order with respect to boric acid concentration if a reaction of complexation occurred. The same researchers mentioned earlier in an EPRI report [9] that a bidendate complex could be the cause of these results by analogy with the study of the aluminate complexation by the bis–tris,2,2-bis(hydroxymethyl)-2,2′,2″-nitrilotriethanol used as pH buffer [10]. They suggested the reaction:

1 $$ {\text{Ni}}\left( {\text{OH}} \right)_{2}\,+\,{\text{B}}\left( {\text{OH}} \right)_{4}^{ - } \rightleftharpoons {\text{Ni}}\left( {\text{O}} \right)_{2} {\text{B}}\left( {\text{OH}} \right)_{2}^{ - } + 2{\text{H}}_{2} {\text{O}} $$

but this explanation would be correct only if the complex is very strong, in order to raise the solubility of nickel hydroxide by orders of magnitude.

On the other hand, Shchigol has studied the solubility of the solid nickel orthoborate Ni(BO_2_)_2_·4H_2_O in boric acid medium [11]. The measured solubility was found to increase upon addition of boric acid. To explain that enhancement, the formation of a nickel borate complex in solution was believed to occur as ${\text{Ni}}\left( {{\text{BO}}_{2} } \right)_{3}^{ - }$. However, the study was controverted [12] because the cation balance was not respected. In addition, the solid was not well characterized, the equilibrium state may not have been reached (lack of data), nickel ion complexation by chlorides or hydroxide ions had not been taken into account and the activity coefficient model was not defined. Nevertheless, a recent review of nickel chemical thermodynamics [12] suggests that a neutral complex could be formed based on Shchigol’s experimental data and according to the following reactions:

2 $$ {\text{Ni}}({\text{BO}}_{2} )_{2} \cdot 4{\text{H}}_{2} {\text{O}}_{{\left( {\text{s}} \right)}} \rightleftharpoons {\text{Ni}}^{2 + } + 2{\text{B}}\left( {\text{OH}} \right)_{4}^{ - } $$

3 $$ {\text{Ni}}^{2 + }\,+\,2{\text{B}}\left( {\text{OH}} \right)_{4}^{ - } + {\text{B}}\left( {\text{OH}} \right)_{{3({\text{aq}})}} \rightleftharpoons {\text{NiH}}\left( {{\text{BO}}_{2} } \right)_{{3({\text{aq}})}} + 5{\text{H}}_{2} {\text{O}} $$

Other papers [13, 14] discussed the mechanism of a nickel–borate complexation but they did not use experimental results. Consequently, valuable experimental data are lacking to confirm the existence of a nickel–boron complex. The aim of this study is, therefore, to experimentally study complexation in solution and to determine the associated equilibrium constants at 25, 50 and 70 °C by using a pH-monitoring method and modeling. The second part of the paper is devoted to the characterization of the complex by first principles calculations, supporting its stability in water.

## Proposition of Nickel–Boron Complex Based on Shchigol’s Experimental Data

Before proposing a nickel–boron complex, the speciation of boron needs to be taken into account to highlight which boron species could be relevant as a ligand. The proposed complex must also fit the experimental data obtained by Shchigol [11]. For that purpose, the software CHESS was used as a modelling tool [15]. Data used for all calculations are available in Table 1. Finally, since the complex strength derived from Shchigol’s work depends on the solubility of the solid that was present, it is necessary to experimentally confirm the presence of the complex in solution, independent from the data of Shchigol.

**Table 1:** CHESS parameters used for simulation

Reactions	log_10_ *K*
${\text{B}}\left( {\text{OH}} \right)_{{3\left( {\text{aq}} \right)}} + {\text{H}}_{2} {\text{O}} \rightleftharpoons {\text{B}}\left( {\text{OH}} \right)_{4}^{ - } + {\text{H}}^{ + }$ ^a^	$\log_{10} K = - 36.2605 + \frac{3645.18}{T} + 11.6402 \, { \log }_{10} T + \left( {16.4914 - 0.023917T} \right)\,\log_{10} \left( {\rho w} \right) + \log_{10} \left( {K{\text{w}}} \right)$ with ρw the density of water in cm^3^·ml^−1^
$2{\text{B}}\left( {\text{OH}} \right)_{{3\left( {\text{aq}} \right)}} \rightleftharpoons {\text{B}}_{2} {\text{O}}\left( {\text{OH}} \right)_{5}^{ - } + {\text{H}}^{ + }$ ^a^	$\log_{10} K = - 3.935 + \frac{1780.5}{T} + 0.95183\,\log \left( T \right) + \log_{10} \left( {K{\text{w}}} \right)$
$3{\text{B}}\left( {\text{OH}} \right)_{{3\left( {\text{aq}} \right)}} \rightleftharpoons {\text{B}}_{3} {\text{O}}_{3} \left( {\text{OH}} \right)_{4}^{ - } + {\text{H}}^{ + } + 2{\text{H}}_{2} {\text{O}}$ ^a^	$\log_{10} K = - 6.495 + \frac{3219.1}{T} + 0.95186\log \left( T \right) + { \log }_{10} \left( {K{\text{w}}} \right)$
$4{\text{B}}\left( {\text{OH}} \right)_{{3\left( {\text{aq}} \right)}} \rightleftharpoons {\text{B}}_{4} {\text{O}}_{5} \left( {\text{OH}} \right)_{4}^{2 - } + 2{\text{H}}^{ + } + 3{\text{H}}_{2} {\text{O}}$ ^a^	$\log_{10} K = - 5.031 + \frac{6001.3}{T} - 1.3572\log_{10} \left( T \right) + 2{ \log }_{10} \left( {K{\text{w}}} \right)$
${\text{Ni}}^{2 + } + 2{\text{H}}_{2} {\text{O}} \rightleftharpoons {\text{Ni}}\left( {\text{OH}} \right)_{{2\left( {\text{aq}} \right)}} + 2{\text{H}}^{ + }$ ^b^	$\log_{10} K_{{{\text{s}}0}} = - 0.335 - \frac{5334.26}{T} - 4.2434 \times 10^{ - 2} \times T$
${\text{Ni}}\left( {\text{OH}} \right)_{{2\left( {\text{S}} \right)}} + 2{\text{H}}^{ + } \rightleftharpoons {\text{Ni}}^{2 + } + 2{\text{H}}_{2} {\text{O}}$ ^b^	$\log_{10} K^{\prime}_{{{\text{s}}0}} = - 2.829 + \frac{4320.17}{T}$
${\text{Ni}}({\text{B}}\left( {\text{OH}} \right)_{4} )_{{2\left( {\text{S}} \right)}} + 2{\text{H}}^{ + } \rightleftharpoons {\text{Ni}}^{2 + } + 2{\text{B}}\left( {\text{OH}} \right)_{{3\left( {\text{aq}} \right)}} + 2{\text{H}}_{2} {\text{O}}$ ^c,d^	${ \log }_{ 10} K_{\text{s}} \left( { 2 5^\circ {\text{C}}} \right) \, = { 9}. 2$
$3{\text{B}}\left( {\text{OH}} \right)_{{3\left( {\text{aq}} \right)}} + {\text{Ni}}^{2 + } \rightleftharpoons {\text{NiB}}_{3} {\text{O}}_{4} \left( {\text{OH}} \right)_{{3\left( {\text{aq}} \right)}} + 2{\text{H}}^{ + } + 2{\text{H}}_{2} {\text{O}}$ ^d^	${ \log }_{10} K_{1} = ( - 3424.6 \pm 180.2)/T + \left( {0.028 \pm 0.557} \right)$

The reactions and the associated equations of the equilibrium constant at infinite dilution were taken from ^a^ Palmer et al. [3]; ^b^ the EPRI report [9]; ^c^ this study, fit to Shchigol et al. [11]; ^d^ this study, fit to experimental data given in Table 3

### Boron Speciation

The speciation of boron strongly depends on the chemical medium and the experimental conditions such as pH, temperature, boron concentration, and counter ions. At concentrations of boron higher than 0.01 mol·kg^−1^, different polymerized species, called polyborates, are formed and can even become dominant in solution. Various studies [2, 3, 16–19] have been conducted to understand the mechanisms of formation and the geometric configuration of polyborate species but their diversities make this study complicated and most of the results are controversial. The studies from Palmer et al. [3] and Mesmer et al. [2] about the determination of polyborate formation constants are considered as reliable because of their relatively good agreement and the existence of experimental data. Palmer et al. propose a dataset that includes either a tetraborate or a pentaborate. In this study we did not take into account the pentaborate since there are discrepancies even on its formula in the literature. Therefore, the following reactions of polyborates formation were used:

4 $$ {\text{B}}\left( {\text{OH}} \right)_{{3\left( {\text{aq}} \right)}}\,+\,{\text{H}}_{2} {\text{O }} \rightleftharpoons {\text{B}}\left( {\text{OH}} \right)_{4}^{ - } + {\text{H}}^{ + } $$

5 $$ 2{\text{B}}\left( {\text{OH}} \right)_{{3\left( {\text{aq}} \right)}} \rightleftharpoons {\text{B}}_{2} {\text{O}}\left( {\text{OH}} \right)_{5}^{ - }\,+\,{\text{H}}^{ + } $$

6 $$ 3{\text{B}}\left( {\text{OH}} \right)_{{3\left( {\text{aq}} \right)}} \rightleftharpoons {\text{B}}_{3} {\text{O}}_{3} \left( {\text{OH}} \right)_{4}^{ - } + {\text{H}}^{ + } + 2{\text{H}}_{2} {\text{O}} $$

7 $$ 4{\text{B}}\left( {\text{OH}} \right)_{{3\left( {\text{aq}} \right)}} \rightleftharpoons {\text{B}}_{4} {\text{O}}_{5} \left( {\text{OH}} \right)_{4}^{2 - } + 2{\text{H}}^{ + }\,+\,3{\text{H}}_{2} {\text{O}} $$

The results from Palmer et al. [3] are given in terms of base hydrolysis reactions, while the CHESS thermodynamic database requires reactions involving only “basic/elementary species” (in this case B(OH)_3_, H^+^ and H_2_O). Furthermore, Palmer et al. provided the equations of the equilibrium quotients, which involve additional parameters linked to the ionic strength for a given medium. At infinite dilution, these parameters can be removed and the activity of water is equal to 1. The associated equations of the equilibrium constants were therefore combined with the dissociation of water in order to agree with Eqs. 4–7. Since the ionic strength effect is calculated by CHESS with a model that is not the same as Palmer’s, a small deviation results on the final calculations, but because of its low value in our chemical conditions, we did not observe any significant effect of this inconsistency. The equilibrium constant equations for reactions 4–7 are available in Table 1.

**Fig. 1:**
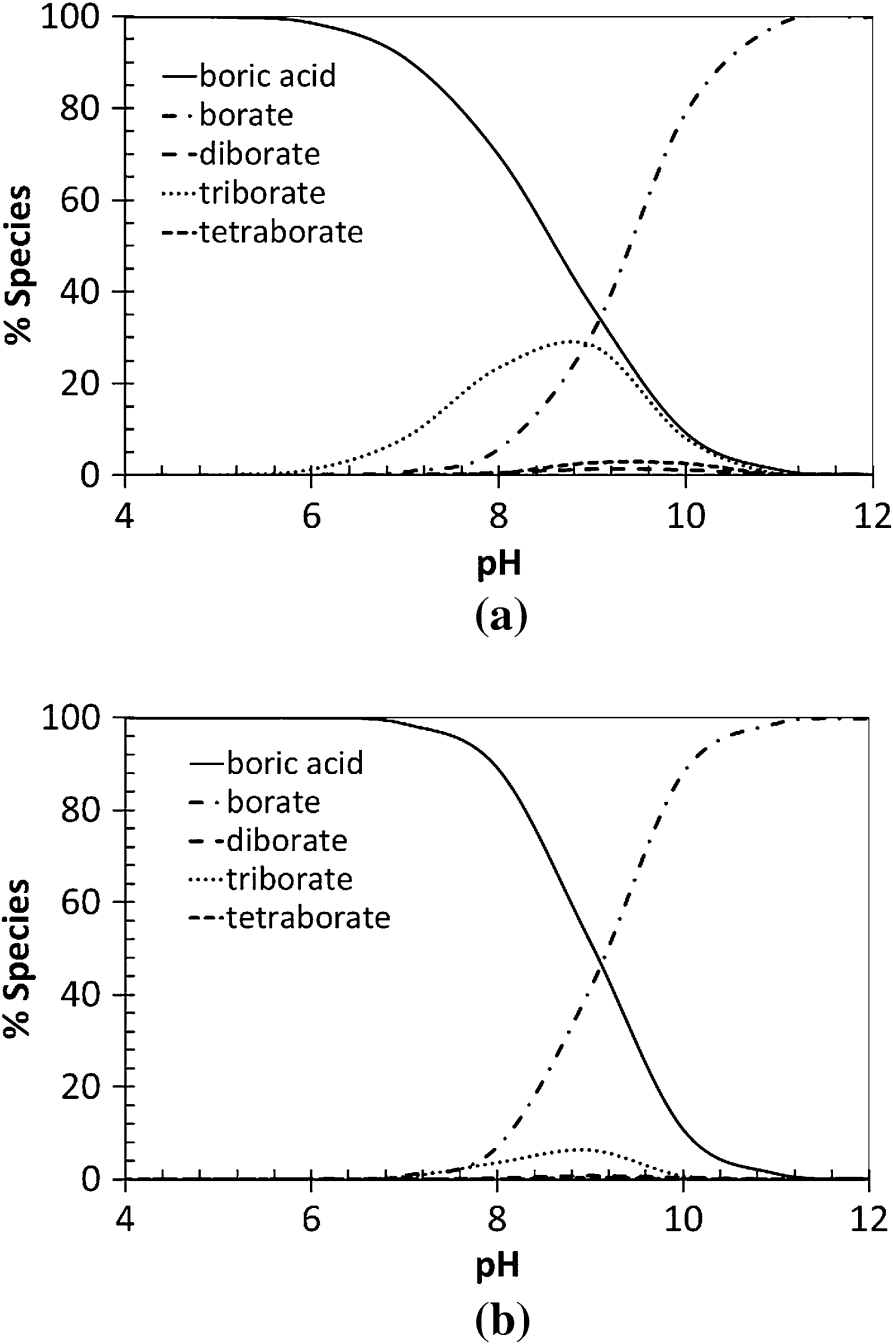
Boron speciation diagram as a function of pH at 25 °C: boron concentration equal to **a** 0.5 mol·kg^−1^, **b** 0.1 mol·kg^−1^

These species are important for understanding the behavior of boron as a function of our chemical conditions. Figure 1 presents the speciation of boron at two different concentrations (A) 0.5 mol·kg^−1^ and (B) 0.1 mol·kg^−1^ and at 25 °C using the equilibrium constants calculated from the fitting equation adapted from Palmer et al. [3] and available in Table 1. It appears that polyborate species exist in solution for pH ranging from 6 to 10. The triborate species is the most abundant polyborate compared to the others and can even become a major species at 0.5 mol·kg^−1^ of boron (Fig. 1a), reaching 35% in solution. In addition, the existence of polyborate species depends also on the total concentration of boron, becoming dominant when the concentration of boron increases to >0.5 mol·kg^−1^. According to Fig. 1b, at 0.1 mol·kg^−1^ of boron, the concentration of the triborate is reduced to less than 10%. Consequently, depending on the chemical conditions, three species are able to lead to the formation of a nickel–boron complex with a quantifiable amount in solution, which are the borate, triborate and boric acid species. Other boron species, such as diborate or tetraborate could nevertheless complex with nickel ions, but regarding to their small concentration (>2% of total boron), it could be difficult to monitor them experimentally.

### Proposition of a Complex

Considering the speciation of boron, the use of high boric acid concentrations, up to 0.7 mol·kg^−1^ and the pH range from 6 to 7.4 in Shchigol’s experiments [11], we can assume that polyborates are abundant under those chemical conditions. Borate ions are minor because of the acidity of the medium. As a consequence, the triborate species is chosen to be a relevant ligand. A neutral complex could be formed, as proposed by Gamsjäger et al. [12] in their review and described in Eq. 8 below:

8 $$ {\text{B}}_{3} {\text{O}}_{3} \left( {\text{OH}} \right)_{4}^{ - }\,+\,{\text{Ni}}^{2 + } \rightleftharpoons {\text{NiB}}_{3} {\text{O}}_{4} \left( {\text{OH}} \right)_{{3\left( {\text{aq}} \right)}}\,+\,{\text{H}}^{ + } $$

The equation can be written differently to fit CHESS thermodynamic database requirements:

9 $$ 3{\text{H}}_{3} {\text{BO}}_{{3\left( {\text{aq}} \right)}} + {\text{Ni}}^{2 + } \rightleftharpoons {\text{NiB}}_{3} {\text{O}}_{4} \left( {\text{OH}} \right)_{{3\left( {\text{aq}} \right)}} + 2{\text{H}}^{ + } + 2{\text{H}}_{2} {\text{O}} $$

with:

10 $$ K_{1} = \frac{{\left[ {{\text{NiB}}_{3} {\text{O}}_{4} \left( {\text{OH}} \right)_{{3\left( {\text{aq}} \right)}} } \right].\gamma_{{{\text{NiB}}_{3} {\text{O}}_{4} \left( {\text{OH}} \right)_{3} \left( {\text{aq}} \right)  }} \times \left[ {{\text{H}}^{ + } } \right]^{2} .\gamma^{2}_{{{\text{H}}^{ + } }} \times a_{\text{w}}^{2} }}{{\left[ {{\text{Ni}}^{2 + } } \right].\gamma_{{{\text{Ni}}^{2 + } }} \times \left[ {{\text{H}}_{3} {\text{BO}}_{3} } \right]^{3} .\gamma_{{{\text{H}}_{3} {\text{BO}}_{{3\left( {\text{aq}} \right)}} }}^{3} }} $$

where *K*_1_ is the equilibrium constant, *a*_w_ the activity of water, and *γ* is the activity coefficient of the species in solution, calculated by the truncated Davies model, which has been preferred over the Pitzer model as, to our knowledge, no coefficients representing the interaction between the nickel cation and the boric acid species are reported in the literature. The most important contribution to the ionic strength value is from the formation of nickel ions, Ni^2+^ ($Z_{i}^{2} = 4$), and not from the acid nor from a salt (such as NaCl) as we did not want to use an electrolyte to fix the ionic strength to a constant value in our experiments. The main reason is that we did not want to introduce some species (Na^+^, Cl^−^) known to form complexes with boron or nickel species. This results in an increase of the ionic strength from 10^−4^ to 10^−2^ mol·kg^−1^ upon the addition of nickel ions to the solution during a trial.

**Fig. 2:**
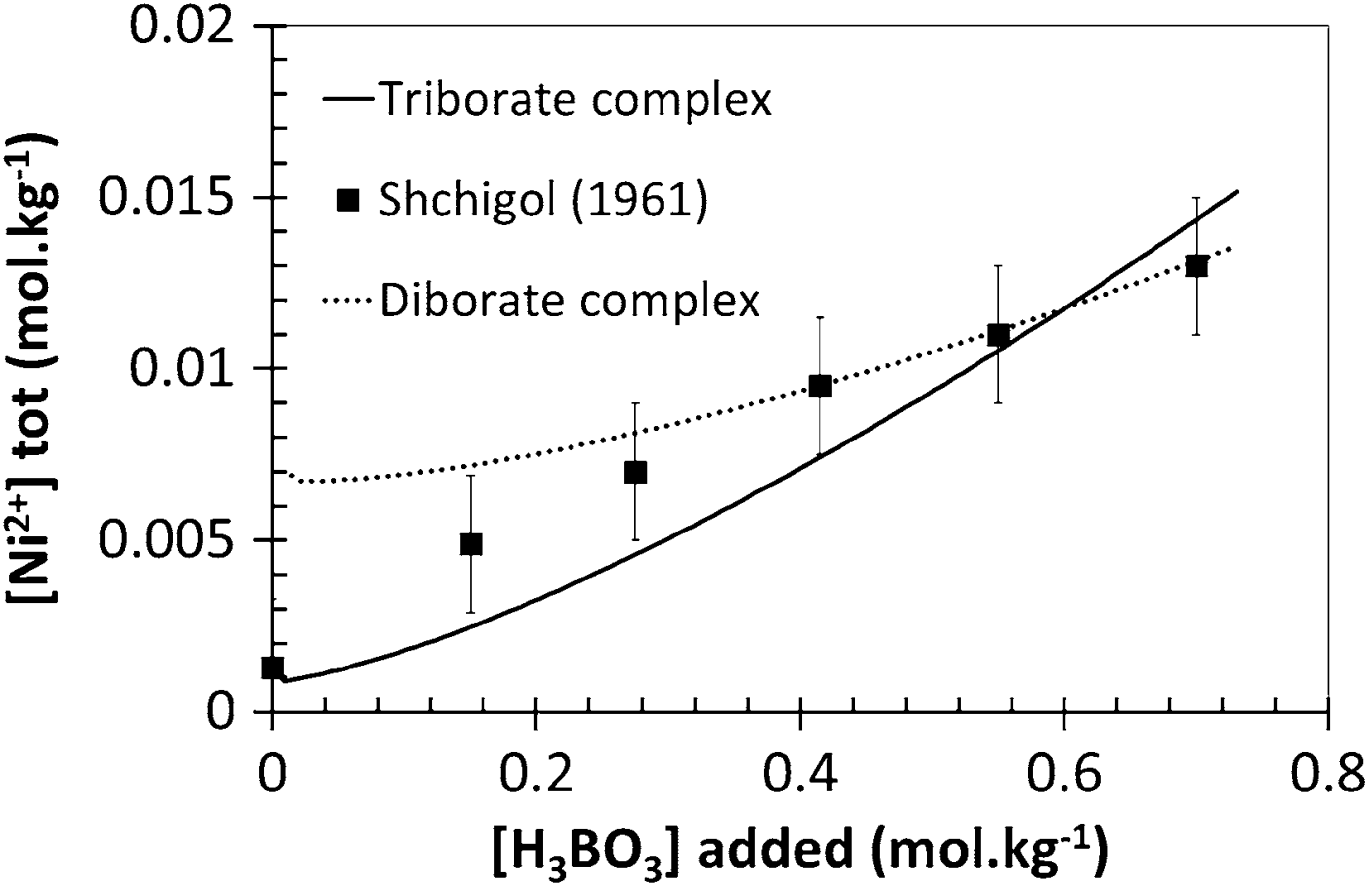
Variation of nickel ions concentration as a function of boric acid concentration, including the formation of a triborate–nickel complex (CHESS speciation model, log_10_ *K*_1_ = −11.1)

**Fig. 3:**
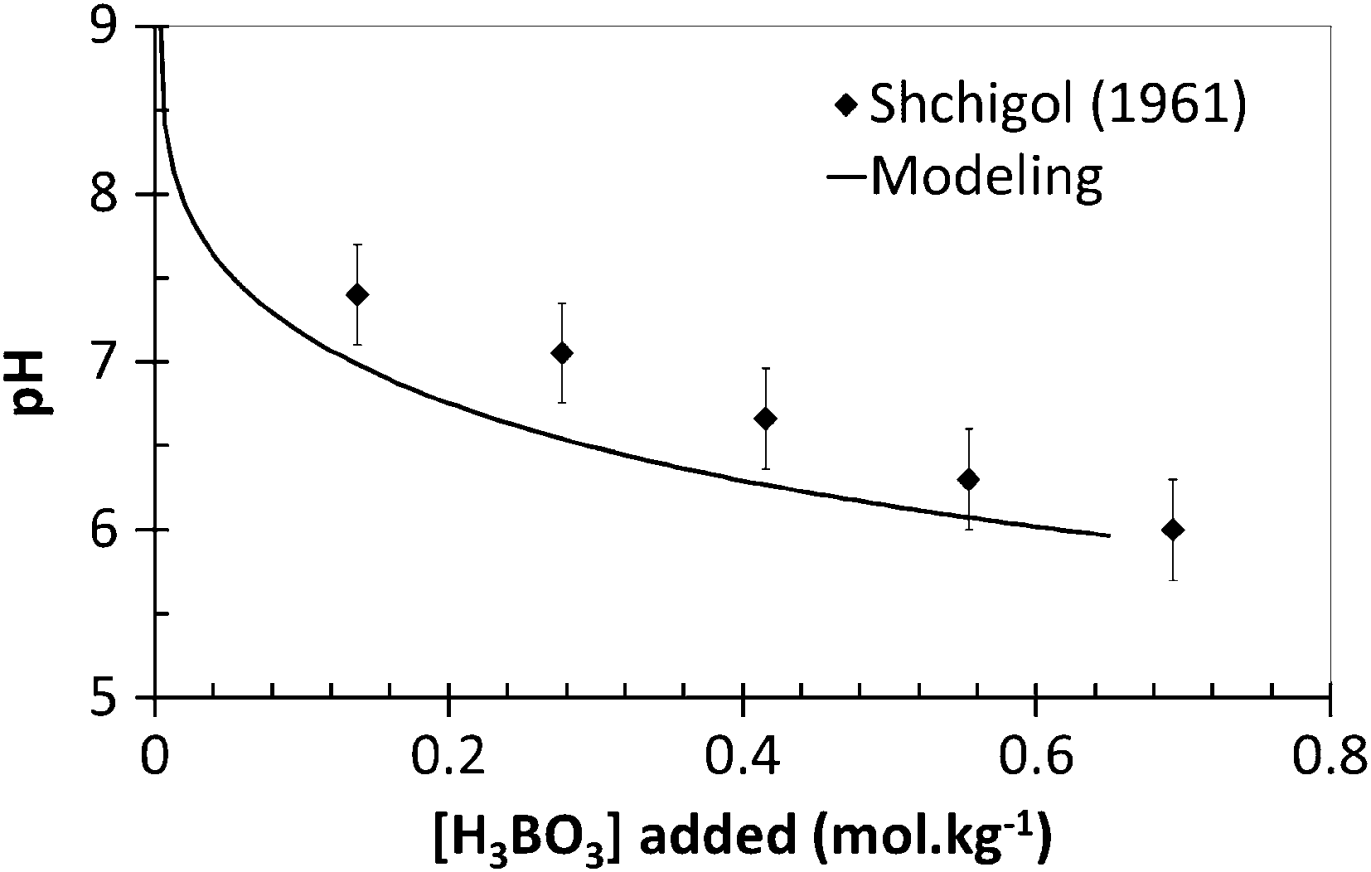
Variation of pH as a function of boric acid concentration including the formation of a triborate–nickel complex (CHESS speciation model, log_10_ *K*_1_ = −11.1)

**Fig. 4:**
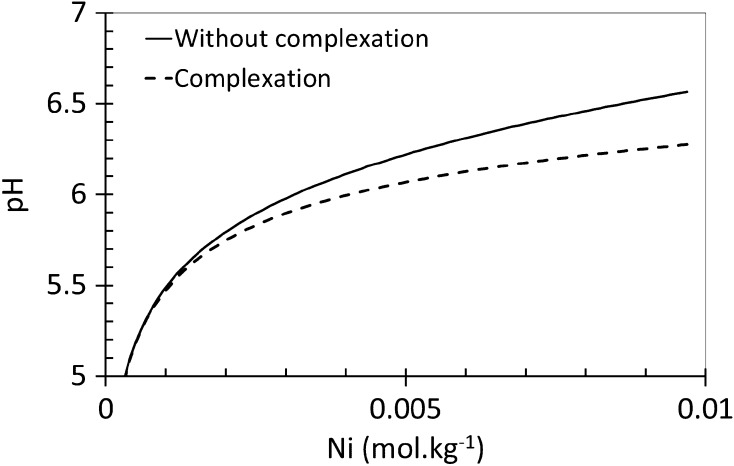
pH variation as a function of Ni^2+^ concentration in boric acid media calculated with CHESS speciation model for a boron solution at 0.5 mol·kg^−1^, initial pH = 4.2, *t* = 25 °C

The solubility of the solid nickel orthoborate with increasing boric acid concentration was simulated with CHESS in order to determine whether the proposed triborate complex would be consistent with Shchigol’s experimental data [11] (the solubility constant of the solid used for these calculations was previously determined by fitting to Shchigol’s experimental data in dilute HCl, being log_10_ *K*_S_ (25 °C) = 9.2 for the reaction reported Table 1). Results of the computation are represented in Fig. 2. The modeling agrees relatively well with experimental data at 0.5 mol·kg^−1^ of boron when log_10_ *K*_1_ is equal to −11.1, but the agreement could be better since a part of the curve is outside of the experimental error bars. Nevertheless, those modeling results were the best and the closest obtained after trying several values of the constant *K*_1_ for the proposed complexation reaction. For a better comparison, further informations is needed on the experimental investigations of Shchigol [11]. To our knowledge, the author did not specify the ionic strength model used and the synthesized solid nickel orthoborate was not characterized. In addition, other potential neutral complexes were tested by following the same simulation protocol, with different ligands such as diborate or borate species, but we did not succeed in obtaining satisfactory agreement between the experimental data and the modeling. Table 2 describes the residual errors associated to the best log_10_ *K* determination obtained between Shchigol’s experimental data [11] and CHESS simulations for the complexation reactions involving these different ligands. These results showed that the smallest residual error, meaning the closest simulation curve to Shchigol’s data [11], was obtained with the triborate simulation. As an example, computation result for a diborate complex is also shown in Fig. 2. It did not fit the experimental data at low concentrations of nickel and the slopes are significantly different. We did not model a complex with a different charge, to be consistent with the assumption of a neutral complex. According to Fig. 3, this hypothesis is also confirmed by modeling the pH of the solution as a function of boric acid concentration, where experimental data and simulation are in quite good agreement, with the assumption of of a triborate complex. Consequently, the proposed reaction of the Ni–B complexation respects the previous enunciated criteria: it is a tri-boron neutral complex, which takes into account the existence of polyborates and also matches reasonably with the experimental data of Shchigol, but this hypothesis must be experimentally confirmed independently from the literature data. According to Eq. 8, a proton is released by the complexation reaction, with an expected impact on pH. The CHESS model presented in Fig. 4 shows pH variations as a function of the concentration of nickel ions in boric acid medium. It has been obtained for a 0.5 mol·kg^−1^ boric acid solution by using the formation constant determined above (log_10_ *K*_1_ = −11.1) as well as the formation constant of polyborates determined by Palmer et al. [3]. It can be seen in Fig. 4 that there is a decrease in pH when the complexation of nickel ions by the triborate occurs. Therefore, it is possible to confirm this model experimentally by pH-monitoring.

**Table 2:** Residual errors obtained between the experimental data of Shchigol [11] and CHESS simulations of the solubility of nickel orthoborate with increasing boric acid concentration, involving different Ni–B complexation reactions

Complexation reactions	Best simulated log_10_ *K*	Residual error
${\text{Ni}}^{2 + } + {\text{B}}\left( {\text{OH}} \right)_{{3\left( {\text{aq}} \right)}} \rightleftharpoons {\text{NiBO}}_{2} \left( {\text{OH}} \right)_{{\left( {\text{aq}} \right)}} + 2{\text{H}}^{ + }$	−16.6	1.6 × 10^−4^
${\text{Ni}}^{2 + } + 2{\text{B}}\left( {\text{OH}} \right)_{{3\left( {\text{aq}} \right)}} \rightleftharpoons {\text{NiB}}_{2} {\text{O}}_{3} \left( {\text{OH}} \right)_{{2\left( {\text{aq}} \right)}} + 2{\text{H}}^{ + } + {\text{H}}_{2} \rm O$	−11.5	3.8 × 10^−5^
${\text{Ni}}^{2 + } + 3{\text{B}}\left( {\text{OH}} \right)_{{3\left( {\text{aq}} \right)}} \rightleftharpoons {\text{NiB}}_{3} {\text{O}}_{4} \left( {\text{OH}} \right)_{{3\left( {\text{aq}} \right)}} + 2{\text{H}}^{ + } + 2{\text{H}}_{2} \rm O$	−11.1	1.4 × 10^−5^

## Experimental and First Principles Approaches to Study the Nickel–Boron Complexation

### Experimental Approach

**Fig. 5:**
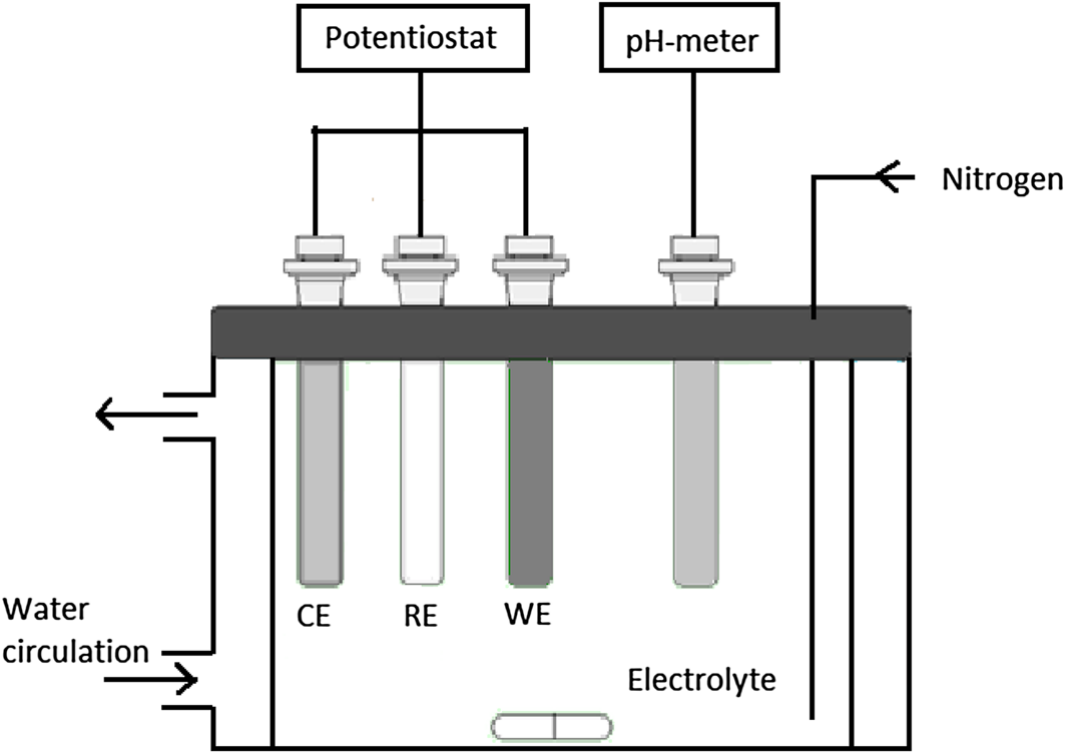
Schematic of the double wall reactor with potentiostat and pH-monitoring, WE is the nickel metal working electrode, CE is the platinum counter electrode and RE is the saturated calomel reference electrode

An experiment based on electrochemical reactions and pH monitoring was performed in a double wall reactor. Nickel ions were gradually formed by oxidation of a nickel metal electrode in a solution of boric acid. The apparatus, schematically represented in Fig. 5, was constituted of four different electrodes. Nickel ions come from a nickel metal electrode (WE) used as anode (Goodfellow, purity 99.98%, 1 mm in diameter, surface of 25 cm^2^). The oxidation reaction is:

11 $$ {\text{Ni}} \to {\text{Ni}}^{2 + } + 2{\text{e}}^{ - } $$

The counter electrode (CE, cathode), where the reduction reaction occurred, is a platinum wire from Radiometer Analytical. The reaction of reduction is:

12 $$ 2{\text{H}}^{ + } + 2{\text{e}}^{ - } \to {\text{H}}_{2} $$

A saturated calomel electrode from Radiometer Analytical was used as reference (RE) and the pH was measured by a combined Ag/AgCl glass electrode from VWR as a function of time and nickel concentration using a Metrohm 780 pH meter. The electrode was calibrated with buffers at pH values of 4, 7 and 10 (from Metrohm) before each experiment. The electrode was also checked after the experiment to ensure that no potential deviation higher than the pH uncertainty had occurred. The nickel electrode was polished with a 1000 grid paper and endured a reduction cycle at −1.3 V versus SCE during 5 min before each test, to avoid the presence of NiO at the surface. The solution was continuously bubbled with nitrogen and the temperature was controlled within ±1 °C by a thermostat. According to the nickel Pourbaix diagram, nickel ions are formed in the potential range −0.2 to 0.8 V versus the hydrogen electrode (1 < pH < 7). The current was fixed at 2 mA by galvanostatic control during each experiment. This value was chosen after running several tests: the higher the current the faster the nickel ions are formed in solution. For values higher than 2 mA, the potential was out of the limits of the Ni^2+^ formation domain, whereas lower values lead to long run times (>48 h). Furthermore, the ionic strength was not fixed by salt addition, to avoid complexation with any cations other than nickel. For that reason, the impedance of the electrochemical circuit was high but this did not disturb the process of nickel ion formation in solution. All the collected data were calculated from the species activities determined by the CHESS modeling with the truncated Davie’s model as mentioned earlier. Results were obtained at 25, 50 and 70 °C.

For each temperature, runs were performed at concentrations 0.5 mol·kg^−1^ of boric acid. Lower concentrations of boron increase the working pH range during a run. This leads to precipitation of nickel hydroxide according to its solubility diagrams. Boron solutions were obtained from boric acid powder (Alfa Aesar puratronic^®^ 99.9995% purity) and concentrations were checked by titration with NaOH. All solutions were prepared from MilliQ water. During each run, 2 mL of the solution were periodically sampled, then filtered (0.2 µm cellulose acetate filter) to analyze the concentration of nickel by ICP–MS (Varian 820 MS).

### First Principles Approach

First principles calculations have been performed in order to determine different properties of the nickel–boron complexes using the NWCHEM code [20]. Their equilibrium stoichiometries and their formation’s energies with respect to the reactions of complexation have been investigated at the B3LYP level [21, 22]. The “solvation model based on density” (SMD) method [23] was used to take into account the solvent effect. Regarding the basis set used to describe the electronic structure of the various molecules and complexes, Gaussian type functions (GTF) have been employed. The H, B [24] and O [25] atoms have been described with all electron GTF basis sets. Standard basis with 5*s*-111*sp*-1p*, 6*s*-311*sp*-1d* and 8*s*-611*sp*-1d* have been adopted for H, B and O, respectively. For Ni, the 3s, 3p, 3d and 4s have been treated as valence electrons combined with a Hay–Wadt small core pseudopotential as described in the literature [26–28] and a 3111*sp*-311*d* basis set. A full description of the different basis is given in the Appendix.

For the evaluation of the exchange correlation contribution to the density functional, the “xfine” grid, as defined in the manual of NWCHEM [20], was used. The convergence criterion on total energies was 10^−8^ au. Atomic displacements and force thresholds were 1.8 × 10^−3^ and 4.5 × 10^−4^ au, respectively. The precision of the obtained energies of reaction and bond length are 1 × 10^−6^ au and less than 0.01 Å, respectively. With these computational conditions, the obtained data can be considered to be fully converged.

## Results and Discussion

The pH variations as functions of nickel ion concentration were obtained at three different temperatures (25, 50 and 70 °C). Experimental data are available in Table 3. Nickel concentrations were an average of five replicates. The associated uncertainty was obtained for a level of confidence at 95%. During each experiment, a small amount of a black deposit was gradually formed on the counter electrode. Scanning electron microscopy identified the solid as metallic nickel. It is probably obtained by the following reaction:

13 $$ {\text{Ni}}^{2 + } + 2{\text{H}}^{ + } \rightleftharpoons {\text{H}}_{2} + {\text{Ni}}_{{\left( {\text{s}} \right)}} $$

but the reaction can be neglected and does not affect the pH measurements.

**Table 3:** Experimental results of the pH variation obtained as a function of the molality of nickel ions

25 °C			50 °C			70 °C		
Time (min)	pH	log_10_ [Ni] (mol·kg^−1^)	Time (min)	pH	log_10_ [Ni] (mol·kg^−1^)	Time (min)	pH	log_10_ [Ni] (mol·kg^−1^)
0	4.11	−4.15 ± 0.01	0	4.18	−8.35 ± 0.08	0	3.70	−8.26 ± 0.07
8	4.33	−4.32 ± 0.03	5	4.38	−4.50 ± 0.06	7	4.20	−4.21 ± 0.04
20	4.52	−4.05 ± 0.02	20	4.59	−4.08 ± 0.04	28	4.45	−3.87 ± 0.05
35	4.73	−3.82 ± 0.04	60	4.91	−3.70 ± 0.04	57	4.75	−3.65 ± 0.04
55	4.85	−3.65 ± 0.02	269	5.47	−3.07 ± 0.01	223	5.32	−3.20 ± 0.02
126	5.18	−3.30 ± 0.03	373	5.58	−2.85 ± 0.03	372	5.44	−2.91 ± 0.04
230	5.44	−3.04 ± 0.04	1385	5.78	−2.46 ± 0.02	1440	5.68	−2.40 ± 0.01
358	5.61	−2.86 ± 0.05	1828	5.80	−2.36 ± 0.01	1694	5.71	−2.32 ± 0.01
1370	6.03	−2.39 ± 0.02				1748	5.73	−2.30 ± 0.01
1846	6.12	−2.29 ± 0.01				1779	5.73	−2.29 ± 0.01
2743	6.10	−2.27 ± 0.01						

### Determination of the Equilibrium Constants

**Fig. 6:**
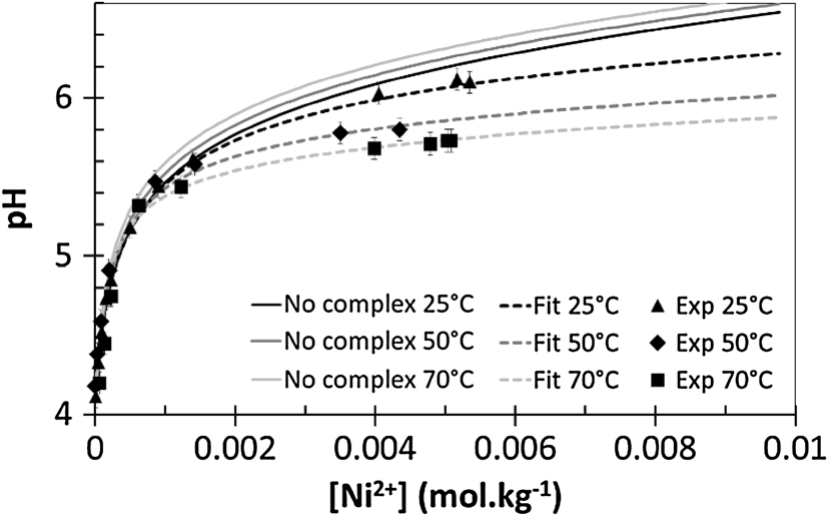
pH variations with increasing nickel ions concentration in boric acid 0.5 mol·kg^−1^. The* solid lines* represent a CHESS speciation model, simulating a media without nickel–boron complexation for the three studied temperatures. The* dashed lines* represent a simulation of the media where nickel–boron complexation occurred

As predicted, the pH increases with the addition of nickel ions to the solution whatever the chemical conditions. However, to demonstrate that complexation occurs, the pH must increase slower than it does in a non-complexing medium. Figure 6 represents the results for an aqueous concentration of boron equal to 0.5 mol·kg^−1^. The “no complexation” curves obtained as a function of the temperature and represented by the solid lines are shown for a better understanding. As we can see, pH variations obtained experimentally are different from those predicted by modeling. The higher the temperature, the less the pH increases as a function of nickel ion concentration. The complexation of nickel ions by the triborate is apparent even at 25 °C. From those results, experimental data where fitted by determining the values of the equilibrium constants. Modeling results are represented by the dotted lines and the values of the calculated equilibrium constants are given in Table 4. Uncertainties were calculated by the partial differential equation method. The most significant contribution to the experimental uncertainties is the pH measurement (pH ±0.05).

**Table 4:** Equilibrium constant of the nickel–boron complex calculated by CHESS simulation at 25, 50 and 70 °C

Complex	log_10_ *K*_1_ (25 °C)	log_10_ *K*_1_ (50 °C)	log_10_ *K*_1_ (70 °C)
${\text{NiB}}_{3} {\text{O}}_{4} \left( {\text{OH}} \right)_{{3\left( {\text{aq}} \right)}}$	−11.50 ± 0.05	−10.50 ± 0.06	−10.00 ± 0.05

At the end of the experiment, the pH was raised to 8 by addition of lithium hydroxide (Alfa Aesar puratronic^®^, 99.99% of purity) in order to observe if a solid precipitates consistent with CHESS calculations that indicate supersaturation for ${\text{Ni}}({\text{BO}}_{2} )_{2} \cdot 4{\text{H}}_{2} {\text{O}}_{{\left( {\text{S}} \right)}}$. After 5 days standing, a green pale precipitate was apparent and was separated from the solution. This precipitate has also been observed by Shchigol, where he postulated the formation of fine crystals of the hexaborate NiB_6_O_10_ from the complex nickel borate solution containing an excess of orthoboric acid and according to:

14 $$ {\text{NiB}}_{3} {\text{O}}_{4} \left( {\text{OH}} \right)_{{3\left( {\text{aq}} \right)}} + 3{\text{H}}_{3} {\text{BO}}_{{3\left( {\text{aq}} \right)}} \rightleftharpoons {\text{NiB}}_{6} {\text{O}}_{{10\left( {\text{s}} \right)}} + 6{\text{H}}_{2} {\text{O}} $$

The deposit was observed by scanning electron microscopy (SEM) and X-ray diffraction (XRD). Agglomerates of thin nanoparticles less than 10 nm in diameter are present. The XRD spectrum shows an amorphous structure partially crystallized. By comparison with solids in the XRD database, several spikes could match with a nickel borate, but nickel hydroxide or bunsenite (NiO) were excluded. Moreover, the atomic composition obtained by energy dispersive X-ray spectrometry shows a quantity of boron 5–7 times higher compared to nickel. As a consequence, the characterization of the precipitate leads to the potential existence of a nickel hexaborate, but other technical approaches must be investigated in order to definitively confirm this hypothesis.

### Influence of Temperature

As can be seen from Table 4, the value of the equilibrium constant increases slightly with temperature. Furthermore, as the complexation reaction, according to Eq. 9, is pseudo-isocoulombic (not strictly isocoulombic as equal numbers of like charged species are not present on either side of the reaction with Δz^2^ = −2), the heat capacity change for the reaction could be small. If we consider the heat capacity change small enough to be neglected, which is often the case for strictly isocoulombic reactions, this assumption allows us to presume that the equilibrium constant is insensitive to pressure changes at constant temperature (<250 °C) [29]. Moreover, this assumption has been used or mentioned in previous studies as for example in the papers of Mesmer et al. [30] and Gu et al. [31], in particular when the pseudo-isocoloumbic reaction contains only positive charges, a very regular temperature dependence can be observed and therefore the heat capacity of the reaction can be assumed to be zero. Thus, a linear equation of log_10_ *K* as a function of temperature is often adequate to describe those reactions over wide ranges of temperature and pressure, meaning that the enthalpy $\Delta_{\text{r}} H^{0} ,$ and the entropy $\Delta_{\text{r}} S^{0} ,$ are constant. The equation of log_10_ *K*_1_ was calculated from the modeling results and leads to:

15 $$ { \log }_{10}  K_{1} = ( - 3424.6 \pm 180.2)/T + \left( {0.028 \pm 0.557} \right) $$

Calculated values of the Gibbs energy change, enthalpy and entropy for the reaction (9) are respectively $\Delta_{\text{r}} G_{{298{\text{K}}}}^{0}$ = (65.4 ± 0.2) kJ·mol^−1^, $\Delta_{\text{r}} H_{{298{\text{K}}}}^{0}$ = (65.6 ± 3.1) kJ·mol^−1^, and $\Delta_{\text{r}} S_{{298{\text{K}}}}^{0}$ = (0.5 ± 11.1) J·K^−1^·mol^−1^. It should be pointed out that the extrapolation of Eq. 15 to temperatures higher than 70 °C must introduce some non-negligible inconsistences, since the reaction is only pseudo-isocoulombic. However, as this work was carried out in order to better understand the behavior of the solid phases of nickel in the PWR primary circuit chemical conditions, an approximation of the thermodynamic data at 300 °C is crucial for further investigations. For example, the results of this study suggest that the complexation reaction significantly enhances the solubility of nickel metal and nickel oxide at pH = 7 and 0.5 mol·kg^−1^ of boron at 300 °C. Furthermore, the complex formation becomes stronger with increasing temperature; meanwhile the concentration of the triborate ligand decreases by depolymerization. The amount of soluble complex could then be lower at high temperature depending on the chemical medium.

The equilibrium constant and Gibbs energy change were also calculated for reaction (8) at 25 °C according to:

16 $$ \log_{10} K_{{{\text{Eq}}.\,8}} = { \log }_{10}  K_{1} - { \log }_{10}  K_{{{\text{Eq}}.\,6}} $$

and are equal to log_10_ *K*_Eq. 8_ = −(4.16 ± 0.05) and $\Delta_{\text{r}} G_{{298{\text{K}}}}^{0}$ = (23.7 ± 0.2) kJ·mol^−1^, respectively. Since the reaction is not isocoulombic (or pseudo-isocoulombic), the enthalpy and the entropy cannot be determined without knowing the heat capacity change.

Other experimental approaches, such as ^11^B NMR or Raman spectroscopy studies, were tested to characterize the complex’s structure, but we did not obtain conclusive results. One explanation is that the concentration of nickel ions is greatly limited by the solubility of nickel hydroxide when the pH is higher than 6, whereas lower pH lead to the disappearance of the triborate species according to the boron speciation. The amount of complex is consequently too low to be efficiently detected by a conventional apparatus. Although further experimental investigations are needed (EXAFS, XANES or UV spectroscopy), a first principle approach has been used in this study to confirm the complex stability.

## First Principles Approach

The simplest way to test the nickel reactivity with boric acid and triborate is to use the first principles approaches to determine the equilibrium geometries and the internal energies of each molecules. Recently, this approach was used by Tossel et al. [32] to calculate absolute p*K*_a_ values for weak acids in aqueous solutions and especially for the boric acid hydrolysis. By carefully using this method, the author showed that it is possible to compare the calculated equilibrium constant with the available experimental data. In this work and for that purpose, the internal energies of the system with the solvent are calculated and simulated with the SMD method [23], taking into account hydration of the molecules in the aqueous system. The internal energy change of the reaction is consequently assumed to be close enough to the Gibbs energy change Δ_r_ *G*_298K_, usually presented in experimental work, regarding the uncertainties and some criteria enunciated in [32]. In the present work, the internal energy change of the following complexation reactions will be approximated as the Gibbs energy change and will be called Δ*G*. They were simulated first in the gas phase but only the results obtained by simulation in the aqueous phase are comparable to our experimental data at 25 °C.

Two different formation reactions of nickel complexes, described by the Eqs. 8 and 9, have been explored: for Eq. 8, the interaction of the nickel with existing triborate reaction R_1_; for Eq. 9, the interaction of nickel with boric acid reaction R_2_.

Since at this level the H^+^ total energy is null (no electron), two ways of treating the energies of reactions 8 and 9 have been explored. The first one called C1, where the experimental value of the H^+^ energy of solvation (−1125 kJ·mol^−1^ as defined in references [32, 33]) has been used, gives the reactions Gibbs energy changes Δ*G*_R1_and Δ*G*_R2_ for Eqs. 8 and 9, respectively:

17 $$ \Delta G_{{{\text{R}}1}} = E_{{{\text{NiB}}_{3} {\text{O}}_{4} \left( {\text{OH}} \right)_{{3\left( {\text{aq}} \right)}} }} - E_{{{\text{B}}_{3} {\text{O}}_{3} \left( {\text{OH}} \right)_{4}^{ - } }} - E_{{{\text{Ni}}^{2 + } }} - 1125. $$

18 $$ \Delta G_{{{\text{R}}2}} = E_{{{\text{NiB}}_{3} {\text{O}}_{4} \left( {\text{OH}} \right)_{{3\left( {\text{aq}} \right)}} }} + 2E_{{{\text{H}}_{2} {\text{O}}^{{}} }} - 3E_{{{\text{H}}_{3} {\text{BO}}_{3} }} - E_{{{\text{Ni}}^{2 + } }} - 1125. $$

For the second one, C2, where H^+^ is replaced by H_3_O^+^ in order to determine Δ*G*_R1_ and Δ*G*_R2_ fully theoretically, the Eqs. 8 and 9 have then to be adapted. The resulting Eqs. 19 and 20 are given below and correspond to the reactions R_1_ and R_2_, respectively:

19 $$ {\text{B}}_{3} {\text{O}}_{3} \left( {\text{OH}} \right)_{4}^{ - } + {\text{Ni}}^{2 + } + {\text{H}}_{2} {\text{O }}\overset {{\text{R}}_{1} } \rightleftharpoons {\text{NiB}}_{3} {\text{O}}_{4} \left( {\text{OH}} \right)_{{3\left( {\text{aq}} \right)}} + {\text{H}}_{3} {\text{O}}^{ + } $$

20 $$ 3{\text{H}}_{3} {\text{BO}}_{{3\left( {\text{aq}} \right)}} + {\text{Ni}}^{2 + }\overset {{\text{R}}_{1} } \rightleftharpoons  {\text{NiB}}_{3} {\text{O}}_{4} \left( {\text{OH}} \right)_{{3\left( {\text{aq}} \right)}} + 2   {\text{H}}_{3} {\text{O}}^{ + } $$

**Fig. 7:**
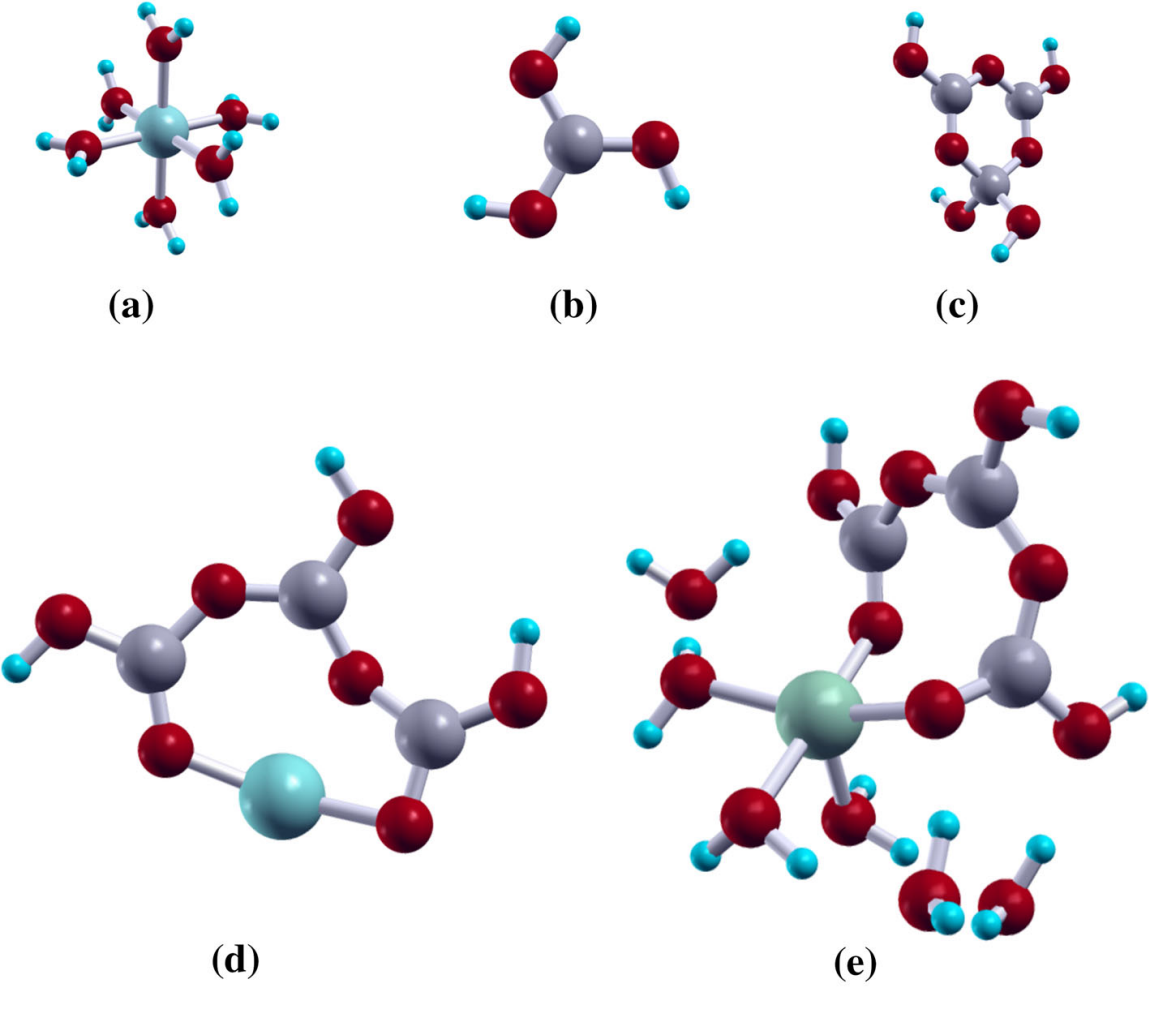
Optimized molecules and complexes at the B3LYP level. **a** Ni^2+^·6H_2_O, **b** H_3_BO_3_, **c** ${\text{B}}_{3} {\text{O}}_{3} \left( {\text{OH}} \right)_{4}^{ - }$, **d** NiB_3_O_4_(OH)_3_ without Ni hydration sphere and **e** NiB_3_O_4_(OH)_3_ with the Ni hydration sphere. The *blue*, *gray*, *red* and *green* atoms are H, B, O and Ni, respectively (Color figure online)

The reactions Gibbs energy changes are then:

21 $$ \Delta G_{{{\text{R}}1}} = E_{{{\text{NiB}}_{3} {\text{O}}_{4} \left( {\text{OH}} \right)_{{3\left( {\text{aq}} \right)}} }} + E_{{{\text{H}}_{3} {\text{O}}^{ + } }} - E_{{{\text{B}}_{3} {\text{O}}_{3} \left( {\text{OH}} \right)_{4}^{ - } }} - E_{{{\text{Ni}}^{2 + } }} - E_{{{\text{H}}_{2} {\text{O}}}} $$

22 $$ \Delta G_{{{\text{R}}2}} = E_{{{\text{NiB}}_{3} {\text{O}}_{4} \left( {\text{OH}} \right)_{{3\left( {\text{aq}} \right)}} }} + 2E_{{{\text{H}}_{3} {\text{O}}^{ + } }} - 3E_{{{\text{H}}_{3} {\text{BO}}_{3} }} - E_{{{\text{Ni}}^{2 + } }} $$

where $E_{\text{X}}$ represents the total energies of molecule X. In this work, these formation energies do not take into account the vibrational and solvent entropies. In order to establish the influence of hydration of the nickel cation, each reaction energy has been determined with Ni^2+^ with or without its first sphere of hydration, and at two levels of approximation: (1) without the solvent (gaseous approximation); (2) with the solvent taken into account via the SMD method. For each of the species, the geometry has been optimized in order to minimize the total energy (their vibrational frequencies have been calculated to verify that the obtained configurations do not correspond to unstable points). For ${\text{B}}_{3} {\text{O}}_{3} \left( {\text{OH}} \right)_{4}^{ - }$, the geometry obtained by Zhou et al. [18] was taken as initial geometry for the optimization. For the NiB_3_O_4_(OH)_3_ complex, the electrostatic potentials around ${\text{B}}_{3} {\text{O}}_{3} \left( {\text{OH}} \right)_{4}^{ - }$ and Ni^2+^·6H_2_O were used to find the best configuration to start the optimization. Figure 7 illustrates the obtained structures. For the different compounds, the geometries are in good agreement with experiments. For instance, the average distances B–O, O–H (in H_3_BO_3_), O–H (in H_2_O), Ni–O (O in the first sphere of hydration) and Ni–O in the complexes are 1.373, 0.965, 0.975, 2.054 and 1.845 Å, respectively, coinciding with the various bond length of aqueous borate and nickel solution, and crystal structures of borate and nickel hydroxides that are reported in the literature [18, 34–36].

The results for Δ*G*_R1_ and Δ*G*_R2_ in the gaseous and aqueous phase are given in Table 5. These data show that, for both reactions C1 and C2, the formation of the complex NiB_3_O_4_(OH)_3(aq)_ is thermodynamically possible. As expected, due to the electrostatic interaction, Ni^2+^ is more reactant with ${\text{B}}_{3} {\text{O}}_{3} \left( {\text{OH}} \right)_{4}^{ - }$ than with boric acid. Δ*G*_R1_ is higher than Δ*G*_R2_. The data illustrate also the solvent effect. Thus the energies in the aqueous phase are approximately five times lower than in the gaseous phase; this is explained by the fact that the interactions between the cation, H_3_BO_3_ and ${\text{B}}_{3} {\text{O}}_{3} \left( {\text{OH}} \right)_{4}^{ - }$ are screened by the dielectric effects and the short range interactions between the solute and the solvent molecules taken into account formally in the SMD method.

**Table 5:** Gibbs energy changes (in kJ·mol^−1^) of the complex NiB_3_O_4_(OH)_3_, Δ*G*_R1_ and Δ*G*_R2_ for the reaction C1 according to the relations 17 and 18 and for the reaction C2 according to the relations 21 and 22, respectively

	Approx.		Δ*G*_R1_	Δ*G*_R2_
Ni^2+^	Gaseous phase	C1	−2074	−1741
		C2	−1677	−936
	Aqueous phase	C1	−376	−296
		C2	−339	−222
Ni^2+^·6H_2_O	Gaseous phase	C1	−997	−664
		C2	−595	141
	Aqueous phase	C1	25	106
		C2	61	179
Experimental (this work)			24	65

Two types of nickel’s environment are explored: without (Ni^2+^) and with its first sphere of hydration (Ni^2+^·6H_2_O). Both reaction are treated in the gaseous and in the aqueous (with the solvent modeled with the SMD method) approximations. The experimental data are given for comparison

The changes are more drastic when the nickel’s first sphere of hydration is treated explicitly in the simulations; then, Δ*G*_R1_ is three times lower than the value obtained without the hydration sphere and the reaction becomes exothermic. The trend in the results is in agreement with the one shown by the experimental data; the R_1_ reaction’s energy is lower than that of R_2_. However, as in references [32, 33], the results show the strong dependence of the reaction energies on the chosen thermodynamic cycle, though the semi-empirical treatment of H^+^ in C1 gives nearest results or results in best agreement with experiment, while the discrepancies with the experiment may be attributed to the complex nature of the reaction. Tossell showed that the precision on the p*K*_a_ determination of the boric acid dissociation depends on the water molecules interacting with B(OH)_3(aq)_ and the formation of ${\text{B}}\left( {\text{OH}} \right)_{4}^{ - }$ [32]. Our approach may need to take into account these types of contributions. Anyway, taking into account the solvent effects, the most thermodynamically favorable complex formation reaction remains R_1_. This result tends to show that the nickel cation in a solute should be more attracted by the existing ${\text{B}}_{3} {\text{O}}_{3} \left( {\text{OH}} \right)_{4}^{ - }$ than with boric acid.

## Conclusion

By modeling previous experimental data of Shchigol [11], we were able to highlight which boron species could be a relevant ligand to complex nickel aqueous species, taking into account the speciation of boron and the chemical conditions. A complexation reaction was proposed where nickel cations react with the triborate, which is the most abundant polyborate species in the pH range between 6 and 10. Furthermore, the experimental pH monitoring data are consistent with the formation of this complex. It appears that the pH is lowered when complexation occurs, starting at 25 °C and to higher extent when temperature is increased to 70 °C. The equilibrium constants were determined at the three temperatures investigated in this study, fitted as a function of temperature and a set of thermodynamics data were deduced. Because we did not succeed in experimentally characterizing the complex by RMN or Raman methods, first principle calculations were used to study its stability. Results of the computations, taking into account the solvent, show that the complexation reaction involving the triborate species is thermodynamically favorable. For all those reasons, we consider the existence of this complex as valid. Nevertheless, it is possible that other complexes between Ni and borate may exist, in particular at lower concentration of boron when polyborates are minor, or with other polyborates and should be further investigated by using, for instance, EXAFS, XANES or UV spectroscopy.

## Appendix

### Atomic Gaussian Basis Sets Used for H, B, O and Ni

All-electron and pseudopotential basis sets have been used for H, B, O and Ni. The B and O all-electron basis sets are the same as used in references [24] for B and [25] for O; for H, the basis set is original. They are contractions of—5*s*-111*sp*-1*p**, 6*s*-311*sp*-1*d** and 8s-411sp-1d*—GTFs for H, B, and O, respectively. For pseudopotential, the Hay–Wadt small-core pseudopotentials [26–28] have been adopted for Ni; the associated basis set are a contraction of—3111*sp*-311*d*—GTFs. The exponents and contraction’s coefficients of the pseudopotential GTFs, as well as for the all-electron basis sets, of the full set of the H basis, of the B-four and O-three outer *sp* and *d* polarization’s shell have been optimized using an energy criterium and are reported in Table 6. The basis set optimization was carried out using the LoptCGscript [37], which performs numerical gradient optimizations based on the conjugate gradient method [38].

**Table 6:** Exponents and coefficients of the contracted Gaussian basis sets adopted in the present study for H, B, O and Ni. For Ni, the basis set is used in conjunction with the Hay–Wadt pseudopotential [26–28]

Atom	Shell	Expt.	Coeff.	
			*s*(*d*)	*p*
H	*s*	157.418362	0.001441	
		92.942487	0.006237	
		17.727431	0.027257	
		9.844505	0.027295	
		3.516237	0.31327	
	*s*	0.904270	1.	
	*s*	0.295470	1.	
	*s*	0.105480	1.	
	*p*	1.074718	1.	
B	*sp*	2.749437	−0.546313	0.171714
		0.589916	1.133410	0.889293
	*sp*	0.360657	1.	1.
	*sp*	0.261831	1.	1.
	*d*	0.684011	1.	
O	*sp*	0.475975	1.	1.
	*sp*	0.161450	1.	1.
	*d*	0.876952	1.	
Ni	*sp*	25.405052	0.004128	−0.043494
		7.273189	−0.608148	−0.115638
		4.162815	0.392273	0.491603
	*sp*	1.633718	1.	1.
	*sp*	0.660162	1.	1.
	*sp*	0.119693	1.	1.
	*d*	50.098640	0.046157	
		14.382472	0.248248	
		4.905609	0.630232	
	*d*	1.672448	1.	
	*d*	0.503344	1.	

For the all-electron basis sets of B and O, only the most diffuse GTFs are given (see Ref. [39] for a complete set of data)
